# Vibration sorting of small droplets on hydrophilic surface by asymmetric contact-line friction

**DOI:** 10.1093/pnasnexus/pgac027

**Published:** 2022-03-16

**Authors:** Yaerim Lee, Gustav Amberg, Junichiro Shiomi

**Affiliations:** Department of Mechanical Engineering, The University of Tokyo, 7-3-1 Bunkyo-ku, Hongo, Tokyo 113-8656, Japan; Department of Mechanics, Linné Flow Centre, The Royal Institute of Technology, SE-100 44 Stockholm, Sweden; Södertörn University, Alfred Nobels allé 7, 141 89 Huddinge, Sweden; Department of Mechanical Engineering, The University of Tokyo, 7-3-1 Bunkyo-ku, Hongo, Tokyo 113-8656, Japan

**Keywords:** wetting, asymmetric contact-line friction, droplet, hydrophilic surface

## Abstract

Droplet spreading and transport phenomenon is ubiquitous and has been studied by engineered surfaces with a variety of topographic features. To obtain a directional bias in dynamic wetting, hydrophobic surfaces with a geometrical asymmetry are generally used, attributing the directionality to one-sided pinning. Although the pinning may be useful for directional wetting, it usually limits the droplet mobility, especially for small volumes and over wettable surfaces. Here, we demonstrate a pinning-less approach to rapidly transport millimeter sized droplets on a partially wetting surface. Placing droplets on an asymmetrically structured surfaces with micron-scale roughness and applying symmetric horizontal vibration, they travel rapidly in one direction without pinning. The key, here, is to generate capillary-driven rapid contact-line motion within the time-scale of period of vibration. At the right regime where a friction factor local at the contact line dominates the rapid capillary motion, the asymmetric surface geometry can induce smooth and continuous contact-line movement back and forth at different speed, realizing directional motion of droplets even with small volumes over the wettable surface. We found that the translational speed is selective and strongly dependent on the droplet volume, oscillation frequency, and surface pattern properties, and thus droplets with a specific volume can be efficiently sorted out.

Significance Statement Dynamic wetting of droplet on a dry solid surface through the action of surface tension is a crucial part of many phenomena in nature and technology, and methods for selectively and directionally controlling motion of the droplet have many potential applications. However, the previous controlling methods using pinning effects have limitation in its efficiency and cannot be applied for small droplets. Here, we show that droplets can be selectively transported at significant speed without pinning on a surface microstructure with anisotropic contact-lines friction. When the surface is shaken at a modest amplitude, droplets in a limited size range travels in a direction predetermined by the microstructure of the surface, allowing for precise motion control and sorting of droplets.

## Introduction

Dynamic wetting, where liquid spreads over a dry surface, is a crucial phenomenon with underlying rich multiphase physics and technological importance in vast industrial processes. Numerous research in the past has made great progress in describing and understanding the general characteristics of dynamic wetting, for instance, in terms of scales and regimes (1, 2). An attraction of the topic lies in the controllability of overall macroscopic flow by the chemistry and physics at the local contact line, where a nonintegrable singularity (3) of the viscous stress makes the detailed nanoscopic features of the liquid and the surface important, giving rise to passive and active controllability with high gain or efficiency involving nonlinear and complex dynamics.

Such controllability is useful in various applications such as spray painting and coating, but is particularly important in microfluidics (4, 5). A common challenge among others is to handle small volumes of liquid, and a useful way to do this is to manipulate small droplets (4), where engineering of the solid surfaces for wetting is a great means. Many different strategies to use the surfaces to control droplet motion have been reported such as using hydrophobic and hydrophilic patterns (6), textured hydrophobic supports in a superhydrophobic surface (7), dynamically controllable microstructure generating a traveling wave (8), and stretchable wrinkled surfaces (9). Another possibility to introduce directionality to the droplet motion is to design anisotropic surfaces, such as nanotextured hydrophobic surfaces patterned with tilted nanorods or nanopillars (10, 11).

Dynamic wetting driven by vibration has been of practical importance and a convenient way to study the contact-line dynamics. The dynamics of a sessile droplet on a vertically oscillating surface is sensitive to the detailed characteristics of the contact line such as magnitude of hysteresis and dissipation (12–14). Furthermore, when asymmetry is induced to the system, a steady droplet motion can be generated. For example, by oscillating an inclined smooth surface or oscillating a horizontal surface in an inclined direction, the droplet can be transported to a specific direction (15–19). Another way to introduce the asymmetry is to use an oscillation waveform that has different accelerations in the forward and the backward part of the droplet motion (20–23). Asymmetry can also be implemented in the surface microstructure, which has an advantage in device implementation being insensitive to the oscillation direction. It has been shown that applying vertical oscillations to a nanotextured hydrophobic surface with tilted nanorods can transport the droplet (11) at speeds on the order of a few millimeters per second when the input frequency is near the natural frequency of droplet oscillation (24). Here, the precise mechanism of the transport is not entirely clear, but it is likely that the one-sided pinning induced by the tilted nanorods play a key role. Pinning forces may allow directional droplet mobility. However, pinning itself acts as a strong energy barrier on contact-line mobility (25), deteriorating the transport speed and restricting the mobility of smaller droplets. Leidenfrost effect is well-known to achieve droplet transport without pinning by levitating the droplet from the solid surface using the superheated solid surface temperature at hundreds of degrees inducing self-vaporized layer in between the droplet and solid surface, but on/off switching and controllability of the droplet motion is difficult to obtain (26–30).

This work aims to explore a possibility to transport water by asymmetric surface microstructure without the pinning effects, with an aim to realize faster and predictable transport. The idea is to utilize contact-line friction that has been previously found to become directional on asymmetric microstructures. In the previous paper, we have performed spontaneous spreading of a droplet on a surface with micron-sized asymmetric sawtooth corrugations and demonstrated that the spreading is faster in the direction to which the contact line moves upward (downward) on the more steeply (gently) sloping face of the ridge (31). The formulated toy model accounting for the detailed shape of the corrugations reproduced the measured spreading speeds and showed that, in our experiments, pinning is not important, but instead the effective contact-line friction plays a dominant role in the mechanism. Here, the contact-line friction means the local dissipation at the contact line caused by the dynamic contact angle being different from the static value, whose rate, as de Gennes described (25), is proportional to ${\mu _f}{U^2}$, where *U* is the contact-line speed and ${\mu _f}$ is the contact-line friction coefficient with the same dimensions as viscosity (denoted ${\mu _l}$ in de Gennes' original paper). This local dissipation can have different hydrodynamic or molecular origins (32–35) and can be effectively modulated by microstructures (36) or electrostatics (37). On the asymmetric sawtooth corrugations, the deviation of the dynamic contact angle from the static value depends on the direction of spreading.

In this paper, by combining the asymmetric contact-line friction and oscillation, we demonstrate a new ratchet mechanism that realizes fast and steady droplet transport, with a droplet placed on asymmetric sawtooth corrugated surfaces and vibrated horizontally. Here, the contact-line friction is an important factor determining the translational speed, and pinning in many cases does not occur achieving faster movement even on rather wettable surfaces. A simple theory is presented that predicts the droplet mean horizontal speed, given the droplet volume and properties, the substrate geometry, and the oscillation frequency. Then, the predicted phenomena are realized by the experiment. It is demonstrated that the fast transport by this pinning-less mechanism, together with the nature of droplet oscillation having the largest amplitude near the resonance frequency, gives rise to efficient vibration sorting of droplets with a specific volume.

### Droplet oscillation system

**Fig. 1.**
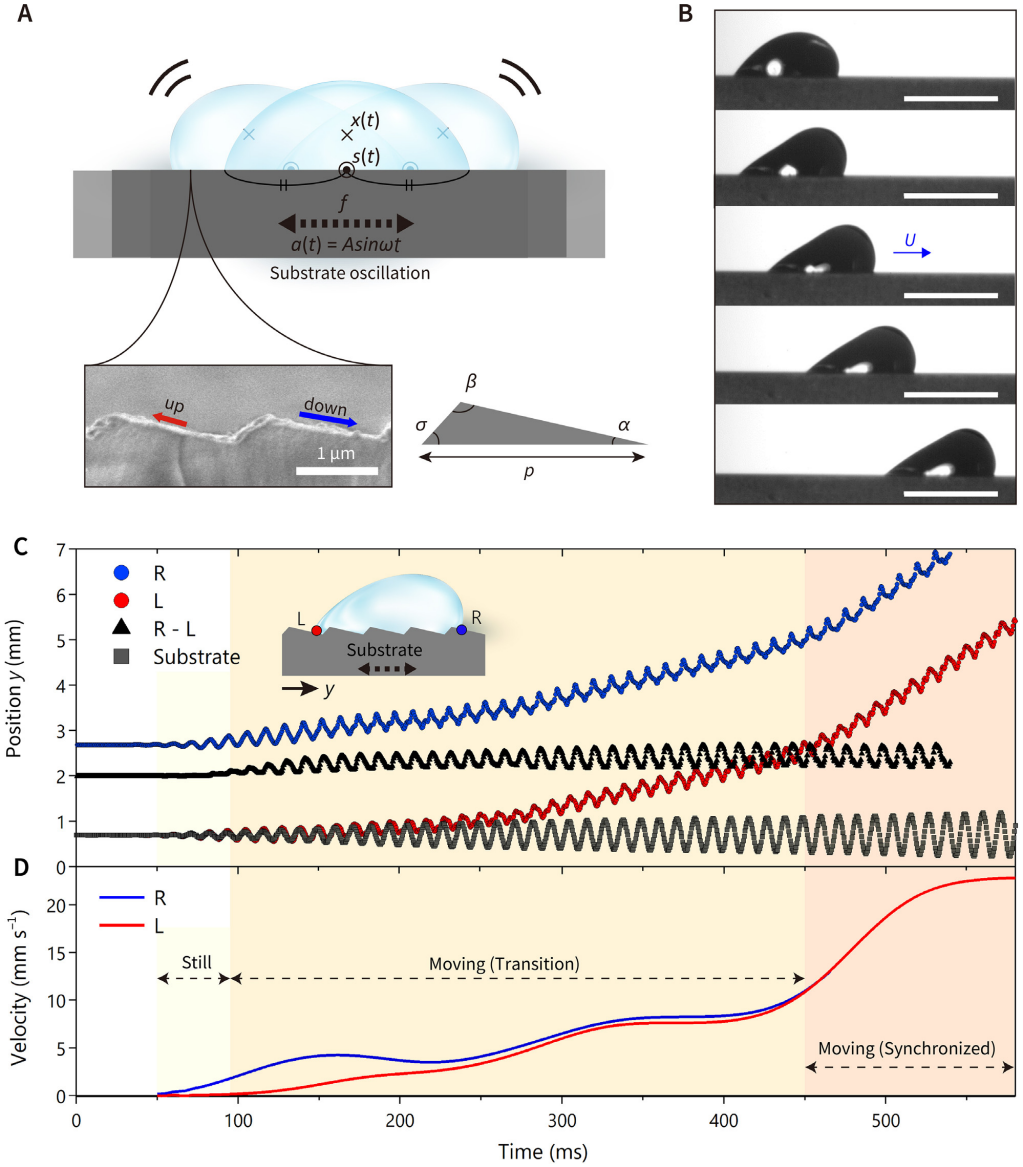
Propulsion of an oscillating water droplet. (A) Schematic of an experimental model showing a water droplet deposited on an asymmetric sawtooth structure oscillating at frequency of *f*. Blowing up shows a scanning electron microscopy (SEM) image of the sawtooth structure with *α* = 13°, *β* = 115°, *σ* = 52°, and *p* = 1.67 μm. The direction (“up” or “down”) is named considering the surface geometry. (B) A water droplet of *V* = 2 μl on substrate oscillated at *f* = 90 Hz starts to move to the down direction, increasing the velocity. High-speed imaging with time interval of 100 ms between the 2 successive images and the bar indicates 2 mm.　(C) Position of designated points (blue circles of R traces the right contact line of droplet and L traces that of the left, black triangles of R–L show droplet footprint length, and gray squares for the position following the substrate motion) on the oscillating substrate with sawtooth surface of *α* = 13°. (D) Analyzed velocity for the R and L points. Refer to Movie S1 (Supplementary Material).

We consider a system with a water droplet of a few microliters placed on a partially wetting substrate surface with micrometer-scale texture of asymmetric shape as shown in Fig. 1(A). Then, the substrate is oscillated horizontally at an input frequency of *f* according to $a\ ( t ) = \ A\ sin\omega t$, where $\omega \ = \ 2\pi f$, and thus excites an oscillatory motion in the droplet. In order to describe the droplet response, we formulate a simple mechanical analog by introducing $x( t )$ to represent the position of the droplet center of mass, and $s( t )$ as the midpoint between the left and right contact lines. We consider a droplet of volume *V* with radius *r*, mass *m*, and approximate height *h* with surface tension *γ*. The following 3 equations constitute a simple dynamic model in terms of the uncompensated Young's stress, a geometric relation, and the equation of motion of the droplet.

(1) \begin{eqnarray*} S{\mu _f}\left( {\dot{s} - \dot{a}} \right)sin\ {\theta _g} = \frac{3}{{2\surd 2}}\ \gamma \left( {cos{\theta _e} - cos{\theta _g}} \right). \end{eqnarray*}

(2) \begin{eqnarray*} \cos {\theta _e} - {\rm{\ }}\cos {\theta _g} = {\rm{\ }} - \left( {s - x} \right)/h. \end{eqnarray*}

(3) \begin{eqnarray*} m\ddot{x} = - 2\gamma r(cos{\theta _e} - \cos {\theta _g}). \end{eqnarray*}

Note that ${\theta _g}$ and ${\theta _e}$ denote a global dynamic contact angle and an equilibrium contact angle, $\dot{s} - \dot{a}$ is the macroscopic contact-line speed relative to the moving substrate. Equation 1 is the same as that derived by Yue and Feng (38) (see their Equation 21, identifying ${\mu _f} = $$\mu \ {\rm{\Pi }}/Cn$ with $\mu $ denoting liquid viscosity and ${\rm{\Pi }}$ and $Cn$ as defined in their paper), with the exception that here the line friction is written as $S{\mu _f}$, which should be interpreted as an effective value for an equivalent flat substrate. ${\mu _f}$ is the contact-line friction coefficient (in units of Pascal seconds, Pa s) on a perfectly flat surface of the same material, which is numerically fitted based on experiments. *S* is a nondimensional geometrical factor that accounts for the increased time for the contact line to pass over the microscopic substrate structures, considering the detailed value of *α, β*, and *σ* as shown in Fig. 1(A) (26, 36).

For the sawtooth shaped asymmetric patterns used here, however, we must expect a directional dependence, this is indeed the essential feature of the problem at hand. Referring to Fig. 1(A), we will, in the following, call a contact line moving to the right (left) as moving in the “down” (“up”) direction and the corresponding *S* value ${S_d}$$( {{S_u}} )$. During one period, the contact lines oscillate left and right, but as the contact line moves faster in the “down”-direction, the net displacement over a cycle is to the right, in the “down” direction. Using this to calculate the distance *L_d_* and *L_u_* traveled on the substrate during the positive and negative half periods respectively, according to Eq. 1 we obtain

(4) \begin{eqnarray*} {\rm{\ }}{L_d} = \frac{\gamma }{{{S_d}{\mu _f}}}\ \frac{3}{{2\surd 2}}\left| \Phi \right|\ \mathop \smallint \limits_0^{T/2} \sin \omega tdt = \frac{{2\gamma }}{{{S_d}{\mu _f}}}\ \frac{3}{{2\surd 2}}\left| \Phi \right|\frac{1}{\omega }, \end{eqnarray*}

(5) \begin{eqnarray*} {\rm{\ }}{L_u} = \frac{\gamma }{{{S_u}{\mu _f}}}\ \frac{3}{{2\surd 2}}\left| \Phi \right|\ \mathop \smallint \limits_{T/2}^T \sin \omega tdt = \frac{{2\gamma }}{{{S_u}{\mu _f}}}\ \frac{3}{{2\surd 2}}\left| \Phi \right|\frac{1}{\omega }, \end{eqnarray*}

where $\Phi $ is the complex amplitude of the oscillation of the contact angle, $\theta_{ g}-\theta_{ e} = \varphi \ ( t ) = {\rm{\ }}re( {\Phi {e^{i\omega t}}} )$. The details of the derivation of Eqs. 4 and 5 are given in the Supplementary Material. Note that we have assumed a hemispherical droplet and will calculate droplet natural angular frequency ${\rm{\Omega }}$ for this (12). Finally, the net translational speed *U* is obtained as the difference between left and right motion, divided by the period of the oscillation, and with the above expression for $| \Phi |$, and using the nondimensional ${\omega _r} = {\rm{\ \omega }}/{\rm{\Omega }}$,

(6) \begin{eqnarray*} U\ &=& \left( {{L_d} - {L_u}} \right)\ \ \frac{\omega }{{2\pi }}\nonumber\\&& = {\rm{\ \ }}\frac{A}{h}\ \frac{\gamma }{{{\mu _f}}}{\rm{\ }}\frac{{\omega _r^2}}{{\sqrt {{{\left( {1 - \omega _r^2} \right)}^2} + \frac{{2\pi }}{3}{{\left( {\frac{{{\omega _r}}}{{O{h_f}}}} \right)}^2}} }}{\rm{\ \ }}\frac{3}{{{\rm{\ }}2\pi \sqrt 2 }}{\rm{\ }}\left( {\frac{1}{{{S_d}}} - \frac{1}{{{S_u}}}} \right). \end{eqnarray*}

Here, $O{h_f} = S{\mu _f}/\sqrt {\rho \gamma r} \ \ $ denotes the effective-line friction Ohnesorge number, using the average of ${S_d}$ and ${S_u}$ for *S* (31, 36, 39). This means that we expect little motion at low frequencies, that the translational speed should be proportional to the oscillation amplitude, and that the maximum speed should occur near the eigenfrequency. The details of the substrate geometry enter through the difference between ${S_d}$ and ${S_u}$, which thus determine the speed.

### Droplet transport

To realize the droplet oscillation system designed in Fig. 1(A), we place a small water droplet with volume of *V* = 2 μl (*r* ≈ 0.78 mm, *γ* = 0.072 N m^−1^, and *μ* = 997 kg m^−^^3^) on an asymmetric sawtooth-shaped structure, which is firmly fixed on the drive arm of a mechanical oscillator. The symmetric sine wave generated by a function generator applied to the substrate via the oscillator. The surface used is coated with a thin aluminum layer on the top with geometry of *α* = 13°, *β* = 115°, *σ* = 52°, and *p* = 1.7 μm (Fig. 1B). The average of *S_d_* ( = 1.22) and *S_u_* ( = 1.59) for *S* is given as 1.40 for the geometry. The top surface was functionalized with APTS((3-aminopropyl) triethoxysilane) to achieve uniform surface wettability with ${\theta _e} = $ 63° and ${\mu _f}$ = 0.09 Pa s, which is obtained from a water droplet on a flat aluminum surface processed with the identical silanization. The above experimental condition gives $O{h_f} = S{\mu _f}/\sqrt {\rho \gamma r} \ \ $= 0.533 close to unity, which means the droplet speed would be influenced by not only the capillary-driven inertia but also the details of the surface geometry. Then the substrate with the droplet was vibrated at *f* = 90 Hz with *A* = 0.5 mm. As we have expected from the theory, we found that the droplet travels to the down direction at a considerable speed of 23 mm s^−1^, which is an unprecedented motion of the small volume of water droplet on the partial wetting surface (Fig. 1B). As we used sawtooth structures of 3 different geometries with *α* of 13, 23, and 26° in the following experiments (Figure S2(a) and (b), Supplementary Material), hereafter we denote each substrate with the values of *α*, as in “*α*13 substrate.”

To understand the detailed behavior and quantitatively analyze the droplet motion, high-speed recordings (Movie S1 and Figure S1(a), Supplementary Material) were digitally processed to track time histories of droplet positions (Fig. 1C). At the interface between the droplet and the substrate, we define the right edge as “R” and the left as “L.” Using the position time histories of R and L from Fig. 1(C), we extracted the velocity of the points as shown in Fig. 1(D). At the start of substrate oscillation (50 ms ∼), the displacements of R and L follow the substrate, and $x( t )$ is almost stationary, named “still” stage. In this stage, only R accelerates towards the “down” direction, due to the deviation of dynamic contact angle from the equilibrium value, while L is temporarily still at the ridge due to contact angle hysteresis. As the vibration amplitude increases beyond a threshold value, the capillary force overcomes the contact-line hysteresis and L also starts moving toward the “down” direction.

As we show in Fig. 1(D), the transition region begins from around 95 ms with the complete motion of the contact line. During this time period of about 355 ms, the droplet cyclically expands and contracts horizontally as seen in the footprint length R–L, gradually elongating the droplet shape along the direction of the oscillation. Simultaneously, the droplet starts to completely slide over the surface as we can see from the time-varying deviation in oscillation phase and the traveled distance per each oscillation between the R or L and the substrate.

We note that the peaks of R and L, are in phase with the substrate at the beginning of the transition stage. However, as the motion develops over time, R finally becomes 180° out of phase. Interestingly, the oscillation direction of $x( t )$ with respect to the substrate is reversed via the transition region taking time to synchronize the motion between the droplet and the substrate. It is noticeable that the droplet can slide over the surface within 45 ms only with the transient substrate amplitude of 0.072 mm, so-called threshold amplitude, and accelerates rapidly within around 120 ms (from 450 to 570 ms) reaching the translational speed once the motion between the substrate and the droplet is synchronized.

Within the synchronized region, $x( t )$ and the substrate oscillate in opposite directions, so that the contact line obtains the maximum possible travel distance with respect to the substrate. The droplet finally reaches a terminal translational speed of 23 mm s^−1^ after about 530 ms from the start of the vibration.

### Frequency dependence

**Fig. 2.**
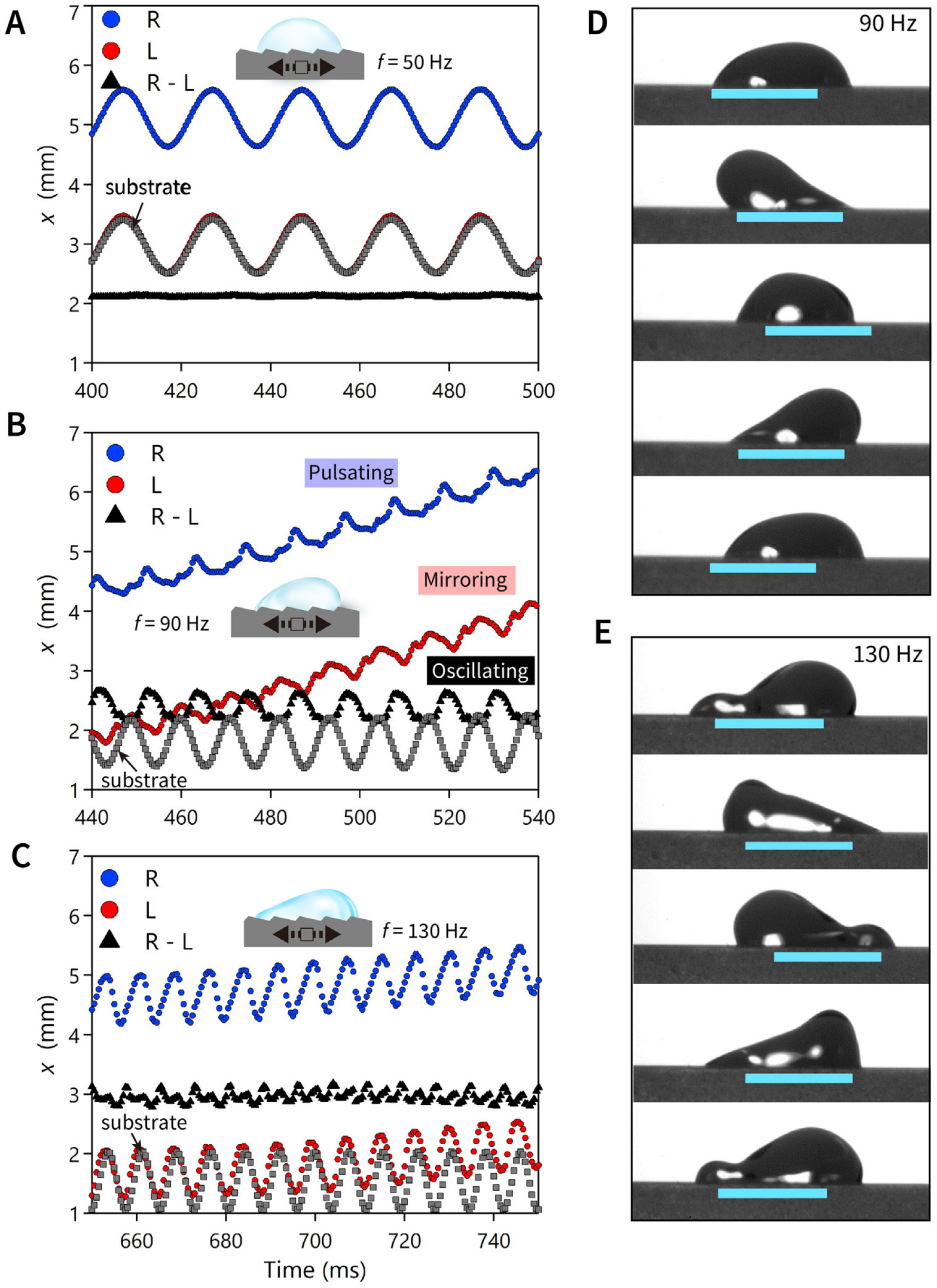
Contact-line motion at different input frequencies. Observed droplet positions at R and L and determined footprint length of R–L for respective frequencies of (A) 50 Hz, (B) 90 Hz, and (C) 130 Hz on the sawtooth surface of *α* = 13° during the time period of 100 ms in the synchronized regime. One cyclic spatiotemporal diagram of the oscillating droplet with oscillation frequency of (D) 90 Hz and (E) 130 Hz with respective time intervals of 2.8 and 1.9 ms. Cyan bars denote 2 mm, tracing the same location on the substrate.

To understand the effect of input frequency, we analyzed droplet behavior as a function of *f* between 10 and 160 Hz, focusing on how the contact-line motion differs and report the representative cases for *f* = 50, 90, and 130 Hz in Fig. 2. In the frequency range between 10 and 50 Hz with *A* of 0.5 mm, a water droplet of 2 μl did not move relative to the sawtooth surface. Figure 2(A) shows the case of *f* = 50 Hz, where L (red circle) and the substrate position corresponding to L at start (gray square) completely overlap with a stationary center of mass with respect to the substrate. As increasing *f*, the droplet started to translate over the sawtooth surface to the “down” direction and the translational speed gradually increased, and the highest translational speed is reached at 90 Hz as shown in Fig. 2(B). The droplet footprint length also oscillates in a sinusoidal manner with an amplitude of 0.25 mm, which is half of *A*. In other words, the droplet oscillation generates an additional harmonic mode at the footprint, which is generally observed when vertical oscillation is applied (13, 40, 41). Interestingly, we observed that the footprint oscillation is accompanied by a pulsating motion of R manifesting the lower contact-line friction to “down” relative to the opposite, which drives $x( t )$ to this direction. The contact line of R moves rapidly during about 1 ms and elongates footprint length temporarily, and the movement speed is close to maximum of the capillary spreading (Figure S3, Supplementary Material). At the same time, L is mirroring R with a 1-ms delay due to the pulsating, which is modest compared to the oscillation period of 11 ms, as seen in the sum of L and R with nearly no oscillation (Figure S4, Supplementary Material).

We show the cyclic droplet behavior in Fig. 2(D) and note how the contact line moves relative to the cyan bar of 2 mm length, tracing the same location on the substrate (refer to Movie S2 [Supplementary Material], which is slower playback speed version of Movie S1 [Supplementary Material]). When the substrate accelerates from the left end and pauses at the right end, moving distance of 2*A* = 1 mm, L is displaced to the left by about 0.5 mm, which corresponds to sliding over 299 pitches to “up.” In the successive half period, R is displaced to the “down” by about 1.25 mm, sliding over 748 pitches to “down.” As the footprint oscillation with amplitude of ∼ 0.25 mm is added on this spontaneous behavior, the ultimate net displacement is ∼ 0.25 mm.

As *f* increases, the droplet oscillation mode evolves and generates more complicated behavior. The droplet remarkably slows down at a higher frequency of 130 Hz as shown in Fig. 2(C) and (E) (see also Movie S3, Supplementary Material). As *f* exceeds the resonance frequency, we expect the oscillation amplitude of $x( t )$ to decrease. Furthermore, when the speed of the substrate $A\omega $ exceeds the capillary line-friction velocity $\gamma /{\mu _f}S$ of 0.57 m s^−1^, the contact line follows the substrate motion closely. We will use the ratio of $A\omega $ and $\gamma /{\mu _f}S$ as a dimensionless quantity to represent line-friction capillary number $C{a_f}$. In the case shown in Fig. 2(E) with *A *= 0.5 mm, both L and R oscillate with amplitudes of 0.4 mm, which means that the droplet is mostly following the substrate. In this case, the line-friction capillary number is $C{a_f}\ ( {\ = {\mu _f}SA\omega /\gamma \ } ) = \ 0.72$, close to unity, thus the contact-line friction limits the velocity difference between the substrate and the contact line. The snapshots at the high frequencies (Fig. 2E) show that the bottom layer of water near the contact line is constrained by the rapidly oscillating substrate, and the upper layer is rather separated from the bottom sustaining its own oscillation mode. Once this footprint has been established, the contact-line motion relative to the substrate is limited and no longer drives the translation of the droplet center of mass. As a result, the elongated footprint length noticeably converges to the value of ${l_0} + 2A$ at the high frequencies, where ${l_0}$ is the initial length of the footprint before oscillation starts (Figure S5, Supplementary Material). The droplet finally stopped to travel at *f* = 140 Hz, with $C{a_f} = \ 0.77$ almost maintaining the footprint elongation with ${l_0} + 2A$ (see Movie S4, Supplementary Material).

### Large droplet volume and reversed transport direction

**Fig. 3.**
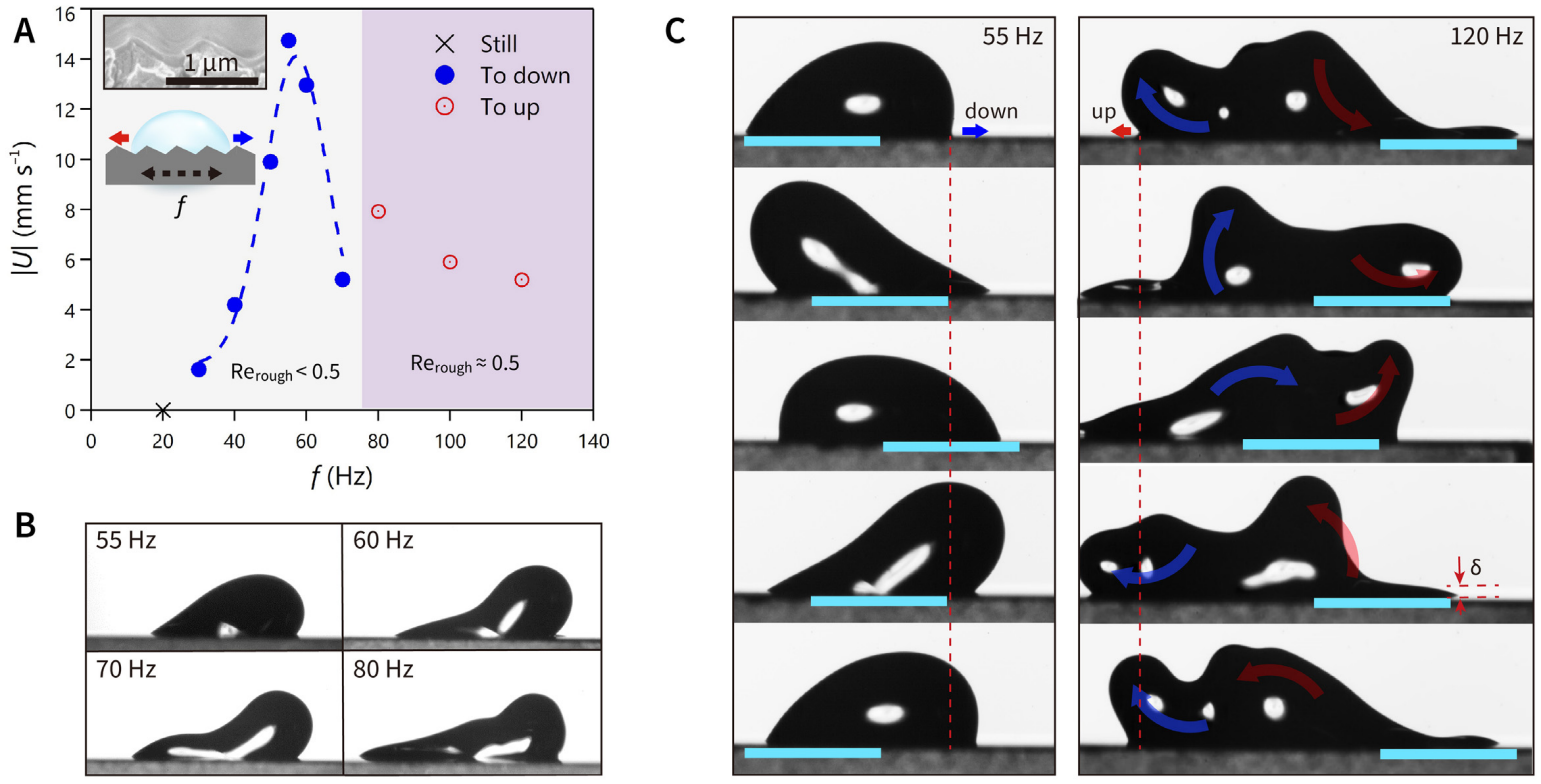
Travel directions of droplet depending on the frequency. (A) Experimentally determined droplet (*V* ≈ 6 μl) translational speed at maximum value vs. driving frequency *f* together with indication of travel direction over sawtooth surface of *α* = 23°. Inset, SEM image of the sawtooth surface structure with *α* = 23°, *β* = 112°, *σ* = 45°, and *p* = 0.82 μm. (B) Snapshots of droplet during travel at the terminal speed. Each image shows the one at a specific time with the highest elongation of footprint length. (C) One cyclic spatiotemporal diagram of the oscillating droplet at frequencies of 55 Hz to downward travel and 120 Hz to upward travel with time interval of 4.5 ms and 2.1 ms, respectively. Red dotted lines in vertical are indicating initial position of leading points depending on the travel directions and colored bars tracking the substrate motions are 2 mm.

To verify how the directional motion differs when the droplet becomes more inertial and surface roughness has lower asymmetry (closer to flat), we evaluated the motion of a larger droplet of *V* = 6 μl (*r* ≈ 1.1 mm) on an asymmetric sawtooth surface with geometry of *α* = 23°, *β* = 112°, *σ* = 45°, and *p* = 0.82 μm (inset of Fig. 3A). *Oh_f_* is given as 0.524 for this case, which is close to the previous *α*13 with *V* = 6 μl case shown in Figs 1 and 2. Note that the structure pitch in this case is half of the *α*13 substrate, thus all the length scales of surface roughness are submicron. Figure 3(A) shows the dependence of droplet translational speed on the input frequency with *A* = 1 mm for the larger water droplet, where the estimated eigenfrequency is 46 Hz. In this case, the droplet starts to travel at *f* = 30 Hz and the translational speed grows with increasing *f* until it reaches the maximum speed of 15 mm s^−1^ at 55 Hz (Fig. 3A and C, Movie S5, Supplementary Material). As *f* exceeds 55 Hz, the droplet gradually elongates (Fig. 3B) and the translational speed drops prominently to 5.2 mm s^−1^ at 70 Hz with corresponding value of $C{a_f}$ = 0.86 (Fig. 3B and Figure S5, Supplementary Material). Interestingly, as *f* exceeds 80 Hz, the droplet alters its moving direction to “up” with apparently elongated footprint. Increasing *f* further, the droplet speed decreases, but remains in the “up” direction. The reversed transport has been observed in the work of Daniel et al., when the droplet size was increased at a fixed input frequency when applying asymmetric waveform on a flat hydrophobic surface (20). They reported transport motion in reversed direction with more inertial droplets in size of nearly 1 or 2 mm in radius and attributed it to the splitting of the droplet oscillation modes. While we agree with the possibility of the mode splitting, we here describe the reversed motion with respect to the emerging liquid layer near the substrate surface accompanied by its movement (see the gradual elongation in Fig. 3B and those changes in tails). Figure 3(C) shows the case of 120 Hz, with extremely complex droplet shape due to higher oscillation modes (Movie S6, Supplementary Material). Here, it becomes clearer how the droplet center of mass is essentially stationary, lubricated by a wetted footprint of length ${l_0} + 2A$, as noted above. Figure 3(C) also shows how a thin liquid film of a thickness similar to a Stokes layer, ${\rm \delta}\sim{( {2\gamma /\rho f} )^{1/2}} \sim 0.1\ mm$, is being pulled out left and right as the substrate oscillates. It is clear from this that the contact-line dynamics has a very limited influence on the net droplet motion, but the motion, rather, is controlled by other weaker effects, such as the finite Reynolds number flow in the Stokes layer over the asymmetric substrate pattern. A representative Reynolds number would be *Re_rough_* = *Aωpρ*/*μ*, where *p* is the pattern pitch. For the case in Fig. 3(C) at *f* = 120 Hz, with *A* = 1 mm, *p* = 0.82 μm, *ρ* = 997 kg m^−^^3^, and *μ* = 0.992 mPa s, *Re_rough_* takes a value of 0.62, which could allow the local flow around the saw tooth ridges to be different going left or right, causing a weak net force in the “up” direction.

### Droplet sorting

It is natural to obtain the maximum droplet mobility at near the eigenfrequency from the droplet oscillation, however, it is not usually easy to realize in a predictable way when the droplet is resting on the solid surface as those liquid–solid interaction generally induces contact-line pinning and additional hydrodynamic effect at inside of droplet, especially when vertically vibrated. From the earlier discussion, we verified that contact-line motion can be continuous over the less than 1 micron scale roughness whose geometry is rather smooth without a sharp edge. If we tune the droplet Ohnesorge number $O{h_f}\ $ and surface fiction in a suitable range where surface geometry can be visible, the anisotropic surface will cause the droplet to move most efficiently at its eigenfrequency, which is sensitive to droplet size. This scenario can realize vibration sorting of droplets with a specific size by asymmetric contact-line friction.

**Fig. 4.**
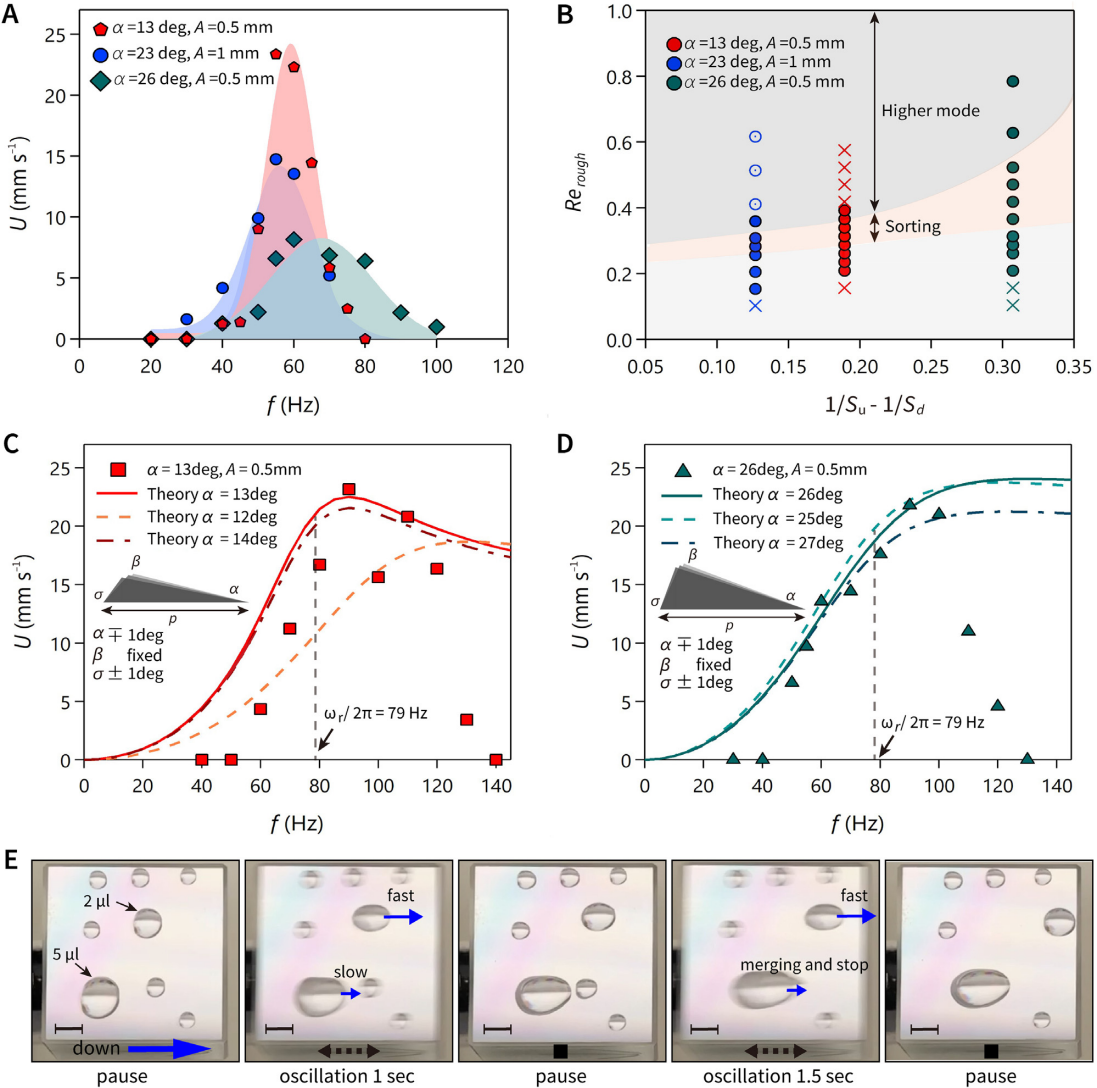
Sorting of oscillating droplets. (A) Translational speed of water droplet over the sawtooth surface depending on the driving frequency with 6 μl droplet. (B) Dynamical behavior of droplets subjected to substrate oscillation described using *Re_rough_* as a function of asymmetry factor of 1/*S_u_*–1/*S_d_*. Coordinates on *x-*axis define the degree of geometry asymmetry positively. Each color of marker gives the specific experimental condition as defined in the legend. The filled circle shows travel to down, the empty circle is to up, and cross is still on the substrate. The orange zone is for droplet sorting region for 6 μl droplet in the frequency region of FWHM along the velocity peaks. The gray zone is higher modes of droplet oscillation with largely elongated footprint length showing dynamic upward, still, and downward motion. (C) and (D) Translational speed of water droplet over the sawtooth surface depending on the driving frequency with 2 μl droplet, respectively for *α* = 13° (C) and *α* = 26° (D) structures. Solid lines show theoretically derived translational speed from Eq. 6 corresponding to each experimental condition. Each dashed line and dash-dotted line illustrates theoretical speed with geometric variations of *α* ∓ 1°, *σ* ± 1° with fixed *β*. (E) Sequence of optical images of the droplets with various volumes resting on the *α* = 13° structure. The substrate is subjected to oscillation at *f* = 90 Hz and *A* = 0.25 mm, repeating with pausing time in between (see Movie S7, Supplementary Material). Scale bars, 2 mm.

To verify the scenario, we traced the droplet behavior for various geometries, and plotted the droplet translational speed *U* as a function of *f* in Fig. 4(A). When the droplet volume is relatively large (*V* = 6 μl), *U* is more sensitive to *f*, showing a sharp peak near the eigenfrequency of 46 Hz. The peaks are sharper in *α*13 and *α*23 cases with full-width half-maximum (FWHM) of ∼ 20 Hz. The maximum obtained speed and the FWHM vary with surface geometry despite the droplet size *R* being the same. Considering the geometry factor as 1/*S_d_*–1/*S_u_* (0.19, 0.13, and 0.31 for *α*13, *α*23, and *α*26 structures, respectively; Table S1, Supplementary Material), the factor closer to 0 makes the sorting range sharper. We report our observation in Fig. 4(B), focusing on the droplet-sorting property. In this diagram, all the experimental parameters of *A, f*, and geometric features of *α, β, σ*, and *p* are included in the presented dimensionless coordinates of *Re_rough_* and 1/*S_d_*–1/*S_u_*. With fixed geometry factor and increasing *Re_rough_*, the droplets can be sorted efficiently within the FWHM range (orange zone). Below the sorting region (white zone), droplets show only feeble travel motion or remain stationary. Above the sorting region (gray zone), the droplet transits to the higher mode and the travel speed is low and the direction may be reversed (*U* < 0) when the surface geometry has a little asymmetry. There exists an optimal range of surface asymmetricity for efficient sorting of droplet as presented with the *α*13 structure showing the narrow FWHM range reaching the highest translational speed of 23 mm s^−1^. For the higher modes obtained for the 6 μl droplet, such as in Fig 3(D) at *f* = 120 Hz, the prediction in Eq. 6 is not applicable since the assumed geometrical relation in Eq. 2 does not describe this complex higher mode of droplet oscillation (Movie S6 and Figure S6, Supplementary Material).

Figure 4C and D show the comparison with the theoretical model in Eq. 6 for the smaller droplet (*V* = 2 μl). Drawn with full solid lines, Eq. 6 is found to agree with the results for *α*13 and *α*26 structures, respectively. Note that the contact line fails to move when the frequency is below 50 Hz (*α*13 case) and 40 Hz (*α*26 case) because the displacement of the center of mass *x*(*t*) and the induced dynamic angle is not enough to overcome the contact angle hysteresis. The droplet in the low-frequency range can be transported by increasing the oscillation amplitude, although the travel speed is lower. We found that geometric variations may sensitively affect the translational speed as shown with the dashed and dash-dotted line, which includes *α* of ∓ 1° and *σ* of ± 1° difference when *β* is fixed (for detail values of *S_d_* and *S_u_* used for the prediction, see Table S2, Supplementary Material). This feature can be used to design the surface structure for faster transport, for example, the transport speed can be increased more than 2 times at the maximum in case of *α*13 structure when the surface shape becomes conventional sawtooth shape with sigma of 90° (see Figure S7, Supplementary Material). Compared to the breakthrough work done by Daniel et al. (20), which actuates drop motion with asymmetric vibration waveform over flat hydrophobic surface generating succession of stop and go motion, our method gives further controllability of the transport as the effective contact-line friction can be tuned by the geometry of the surface structure, which has not been achieved before. We validated possible droplet volume variations from the experiment (sum of systematic and random error of ± 0.07 μl when the droplet volume is 2 μl), but it only merely altered the prediction results and not shown on the graph. In the proposed theory, it should be noted that the model is expected to give qualitatively reasonable predictions for *f* up to and around the first eigenfrequency, but not beyond (see the derivation of model theory in the Supplementary Material). Showing a broader sorting range with FWHM of ∼ 50 Hz compared to that of the larger droplet case, cutoff frequency, where the translational speed drops significantly at 120 Hz for *α*13 and 100 Hz for *α*26, is lower for larger $S\ (\ = \ ({S_d} + {S_u})/2\ = \ 2.5)$ with *α*26 structure, whereas *S* = 1.4 for the *α*13 structure, becoming stationary with *Ca_f_ *≈ 1.1 at 130 Hz.

As the larger volume of droplets can only travel in a narrow-frequency range, droplets lying over the asymmetric sawtooth structures can be sorted in a controlled manner. Figure 4(E) and Movie S7 (Supplementary Material) illustrate what happens when several droplets of different volume resting on the *α*13 structure are oscillated at 90 Hz with amplitude of 0.25 mm. We first oscillate for 1 including the rising and falling time of the vibration and pause, then we oscillate again for 1.5 s in the same way. In this situation, specific droplets (2 μl and 5 μl) move toward “down” direction at different speeds, rather faster with the 2 μl drop, while the 5 μl drop is almost stationary showing apparent elongation. The 5 μl droplet finally stops when it merges with a small droplet of 0.7 μl, while the 2 μl droplet continues to move forward. As shown, the oscillation can turn the droplet travel motion on and off in a controlled manner using the frequency and amplitude. Using the *α*13 structure, we further demonstrate the rapid sorting of a 2 μl droplet at *U* > 20 mm s^–1^ with *f* = 90 Hz and *A* = 0.5 mm (Movie S8, Supplementary Material), sorting of 6 μl droplet at low speed at *U* ∼ 1 mm s^–1^ with *f* = 40 Hz and *A* = 0.5 mm (Movie S9, Supplementary Material).

## Conclusion

We introduced a new approach to directionally transport droplets on vibrated micron-sized asymmetric surface structures. By using a simple model based on fundamental theory of fluid dynamics and droplet motion, we show how the translational speed is determined by the contact-line friction and the detailed surface geometry. With the given structure scale of a micron or less, the substrate smoothly slides under the droplet, and the anisotropic contact-line friction can cause a directional rapid travel on the surface. We identify that the speed is considerable (*U* > 20 mm s^–1^) compared to the reported works (7, 8, 13, 14, 24, 40), and resolve limitation in movable droplet size to a certain extent. We observed that the system can transport water droplets down to 0.7 μl, which was usually difficult to move due to a strong pinning force. This gives rise to large sensitivity to the droplet properties like the size realizing an efficient sorting of droplets with a specific property. This approach can be used without limitation in travel distance and is expected to extend to other liquids with various surface tensions and viscosities (31), realizing systematically predictable and controllable droplet transport and sorting.

## Materials and Methods

### Surface treatment

The surfaces used in the experiment were cleaned by ultrasonic in acetone for 15 min and activated by UV ozone plasma, 10 min prior to the surface functionalization. The activated substrates are enclosed in a Teflon container under nitrogen with 0.1 ml (3-aminopropyl)triethoxysilane (APTS) reagent (obtained from Tokyo Chemical Industry Co., Ltd.) to avoid reaction between the silane and the atmospheric water. The container was heated up to 110° for 15 min in a muffle furnace and the reagent inside was completely evaporated by keeping the temperature for 2 h. The silanization procedure forms a uniform monolayer, and thus does not alter surface structures at micro nor nanoscales. The surface functionalization process showed averaged static contact angle of 63° on the flat aluminum surface sputtered with 100 nm thickness on Si substrate in a clean room from 3-times of independent silanization processes.

### Droplet oscillation experiment

Schematic of the experimental setup of the droplet oscillation is shown in Figure S1 (Supplementary Material). The droplet behavior was observed from the horizontal direction along to the ridges with a high-speed camera (Phantom VEO710L, Vision Research Inc., 6000 fps). A lamp (HVC-SL, Photoron) was used to provide lighting with sufficient intensity. We attached the sawtooth surface substrate on a stage, which was connected to a function generator via an amplifier (Mechanical wave driver SF-9324, PASCO scientific and low-frequency power oscillator URP-20, SHIMADZU). Droplet volume was controlled using a micropipette (Micropipette Research Plus 3120, Eppendorf) and the sum of systematic and random error with absolute value is ± 0.07 μl at 2 μl and ± 0.13 μl at 6 μl estimated from the Eppendorf data file.

### Image processing

The length scale of the image pixel was calibrated using a micro needle with tip diameter of 183 μm, measured using optical microscope (BX51, Olympus), and the distance between the camera lens and the droplet was kept constant. The images taken from the high-speed camera were digital processed using MATLAB to extract the time histories of droplet and substrate position. Approximate height, *h*, value of the droplet in Eq. 2 was extracted from the image using the one before oscillation starts.

## Supplementary Material

pgac027_Supplemental_Files

## Data Availability

All data needed to evaluate the conclusions in the paper are present in the paper and the Supplementary Materials.
