# Supplementary material for: Vibration sorting of small droplets on hydrophilic surface by asymmetric contact-line friction
Source: PNAS Nexus. 2022 Mar 16;1(2):pgac027. doi: 10.1093/pnasnexus/pgac027 (PMC9802364; doi:10.1093/pnasnexus/pgac027)
Supplement: pgac027_Supplemental_Files [file pgac027_supplemental_files.zip › PNASNEXUS-PNASNEXUS-2022-00193-T-s01.pdf]

Vibration sorting of small droplets on hydrophilic surface by asymmetric contact-line friction

Yaerim Lee[1], Gustav Amberg[2,3] and Junichiro Shiomi[1,*]

[1]Department of Mechanical Engineering, The University of Tokyo,
7-3-1 Bunkyo-ku, Hongo, Tokyo, 113-8656, Japan.
[2]Department of Mechanics, Linné Flow Centre,
The Royal Institute of Technology, SE-100 44 Stockholm, Sweden.
[3]Södertörn University, Alfred Nobels allé 7, 141 89 Huddinge, Sweden.

*To whom correspondence should be addressed.

Prof. Junichiro Shiomi
The University of Tokyo
Department of Mechanical Engineering
7-3-1 Hongo, Bunkyo-ku Tokyo 113-8656
JAPAN
Phone: +81(3) 5841-6283
Email: shiomi@photon.t.u-tokyo.ac.jp

Supplementary Information

Derivation of model theory
Fig. S1. Schematic of the experimental setup of the droplet oscillation.
Fig. S2. Surface geometry of sawtooth structure and roughness factor.
Fig. S3. Rapid pulsating motion of droplet.
Fig. S4. Mirroring behavior of oscillating droplet.
Fig. S5. Elongation of footprint length of oscillating droplet.
Fig. S6. Comparison of translational speed with theory for the large droplet volume.
Fig. S7. Comparison of theoretical translational speed with geometric variations.
Tables S1 to S2
Legends for movies S1 to S9

Derivation of model theory

In Eqs. 1-3 in the main text, we introduced a simple dynamic model in terms of Young's stress, the geometric relation, and the motion of the droplet. We copied the Eqs. 1-3 for easier read below.

$$S\mu_f(\dot{s}-\dot{a})sin\theta_g = \frac{3}{2\sqrt{2}}\gamma(cos\theta_e - cos\theta_g) \tag{S1}$$

$$\cos\theta_e - \cos\theta_g = -(s-x)/h \tag{S2}$$

$$m\ddot{x} = -2\gamma r(\cos\theta_e - \cos\theta_g) \tag{S3}$$

Equation S1 is the relation between the uncompensated Youngs stress on the right-hand side, with the global dynamic contact angle θ_g (the angle between the liquid-gas interface and the horizontal macroscopically observed substrate) and the equilibrium contact angle θ_e, and the force resulting from line friction on the left-hand side, as derived from diffuse interface descriptions of the contact line problem. $\dot{s}-\dot{a}$ is the macroscopic contact line speed relative to the moving substrate, μ_f is the contact line friction (in units of Pascal seconds) on a perfectly flat surface of the same material.

S is a nondimensional geometrical factor that accounts for the increased time for the contact line to pass over the microscopic substrate structures. For patterns with high symmetry, such as square posts or a sinusoidal shape, S can be shown to be approximately given by the ratio of the wet area to the projected footprint area of a substrate structure.

Equation S2 expresses the geometrical relation between the dynamic contact angle θ_g and the simplified description of the droplet in terms of the position of the droplet center of mass and the position of the droplet footprint. Equation S3 gives the mass times acceleration of the droplet equated to the net forces acting on the droplet, disregarding the viscous shear stress over the droplet footprint. Making the approximation that the deviation from equilibrium is modest, i.e., $\varphi = \theta_g - \theta_e \ll 1$, and eliminating the angle, Eqs. S1 - S3 can be simplified to

$$m\ddot{x} = 2\gamma r(s - x)/h \tag{S4}$$

$$\dot{s} - \dot{a} = -\frac{2\sqrt{2}}{3\sin\theta_e}\frac{\gamma}{S\mu_f}\frac{s - x}{h} \tag{S5}$$

Putting $x(t) = re(Xe^{i\omega t})$, $a(t) = re(Ae^{i\omega t})$, $s(t) = re(\Sigma e^{i\omega t})$ the solution is readily obtained as

$$X = A\frac{1}{1 - \omega_r^2 + i\omega_r\zeta} \tag{S6}$$

where $\omega_r = \omega/\Omega$, $\Omega = \sqrt{\frac{2\gamma r}{mh}}$, $\zeta = \frac{1}{\tau_f\Omega}$, $\tau_f = \frac{hS\mu_f}{\gamma}\frac{2\sqrt{2}}{3\sin\theta_e}$. Note that $\zeta = \frac{3\sin\theta_e}{2\sqrt{2}}\sqrt{\frac{2\pi}{3}}\frac{\sqrt{\rho\gamma r}}{S\mu_f} = \frac{3\sin\theta_e}{2\sqrt{2}}\sqrt{\frac{2\pi}{3}}\frac{1}{Oh_f}$, with $Oh_f = \frac{S\mu_f}{\sqrt{\rho\gamma r}}$. As expected, the droplet center of mass will move with the substrate at frequencies below the eigenfrequency ($\omega_r < 1$) and become essentially stationary at high frequencies.

This finally gives an estimate of the amplitude in the dynamic contact angle $\varphi = \theta_g - \theta_e$, by substituting in Eq. S3, expanding for $\varphi \ll 1$, and using Eq. S6:

$$\Phi = \frac{A}{h}\frac{\omega_r^2}{1 - \omega_r^2 + i\omega_r\zeta} \tag{S7}$$

where Φ is the complex amplitude $\varphi(t) = re(\Phi e^{i\omega t})$.

The geometrical factor S has up to this point been assumed to be independent of the direction of the contact line motion. For the sawtooth shaped asymmetric patterns used here however, we must expect a directional dependence, this is indeed the essential feature of the problem at hand. Referring to fig 1b, we will in the following call a contact line moving to the right (left) as moving in the 'down' ('up') direction, and we will call the corresponding S values S_d and S_u. For droplet spreading, S_d and S_u were calculated for the sawtooth shapes used here, see Eqs. S9 and S10. The essential finding there is that the spreading is generally faster in the 'down' direction, i.e., $S_d < S_u$. For

the basic droplet oscillation described by Eqs. S6 and S7, we will use $S = (S_d + S_u)/2$ where it enters in the definition of ζ.

The droplet is thus oscillating from left to right, but due to the modest difference between S_d and S_u we expect it to move slightly further in the 'down' direction during its leftward motion, than in the 'up' direction when moving right, and thus to have a net motion towards the 'down' direction. Writing the dynamic contact angle deviation from equilibrium as

$$\varphi(t) = \theta_g(t) - \theta_e = |\Phi| \sin \omega t \qquad \text{(S8)}$$

Using this to calculate the distance traveled on the substrate during the positive half periods for L_d and the negative half periods for L_u, according to Eq. S1 we obtain Eq. 4 and 5 as described in the main text. And finally, the net travel speed of the droplet is obtained in Eq. 6.

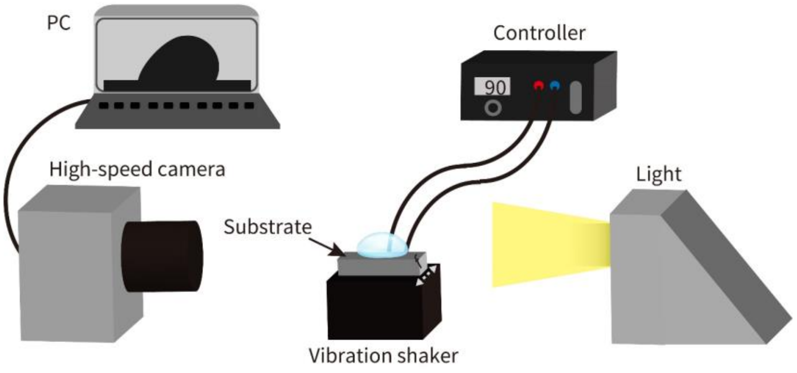

Figure S1. Schematic of the experimental setup of the droplet oscillation. The droplet behavior was observed from the horizontal direction along to the ridges with a high-speed camera (Phantom VEO710L, Vision Research Inc., 6000 fps). A lamp (HVC-SL, Photoron) was used to provide lighting with sufficient intensity. We attached the sawtooth surface substrate on a stage which was connected to a function generator via an amplifier. The images taken from the high-speed camera were digital processed to extract the time histories of droplet position.

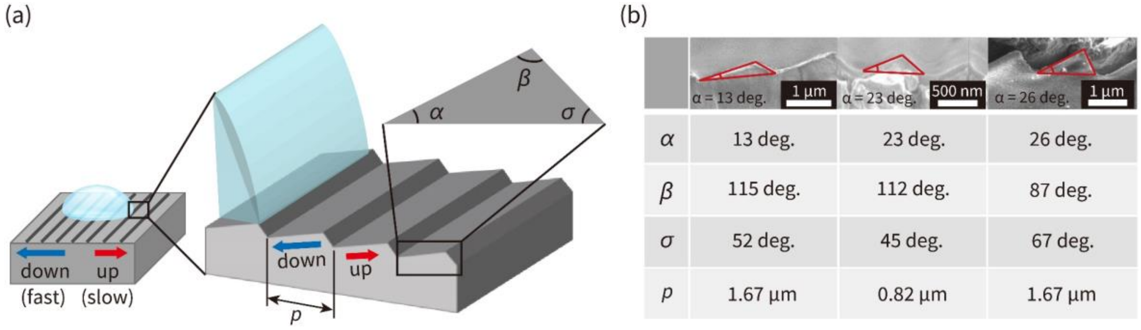

Figure S2. (a) Magnified view of structure surface with droplet showing definition of moving directions and geometry. (b) Detailed geometry values of the sawtooth surface with corresponding cross section view of scanning electron microscopic images.

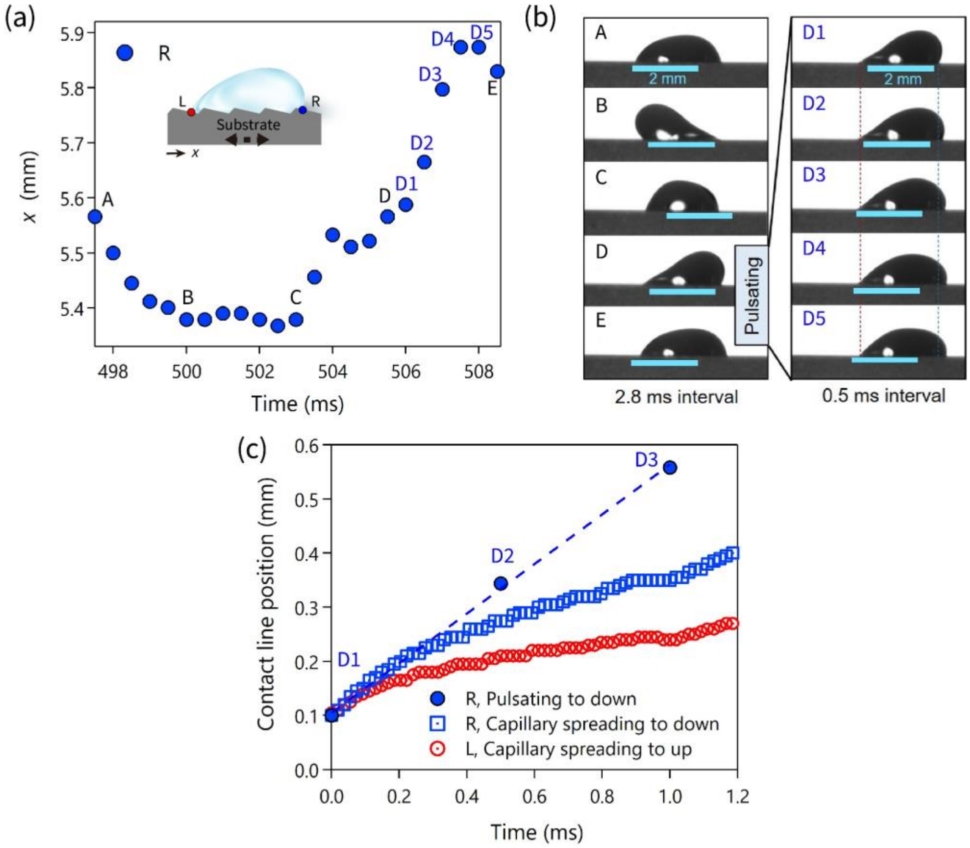

Figure S3. (a) Observed droplet positions at R during one cycle of substrate vibration at 90 Hz in the synchronized region between 297.5 ms and 508.6 ms. (b) One cyclic spatiotemporal diagram of the oscillating droplet corresponding to the notations of A-E in (a) on the left column. Pulsating behavior of droplet to R (down) direction to the surface geometry on the right column corresponding to the notations of D1-D5 in (a). (c) Comparison of contact line moving between capillary spreading of droplet in early time region and pulsating (relatively to the substrate position, $|s\text{-}a|$) during droplet oscillation.

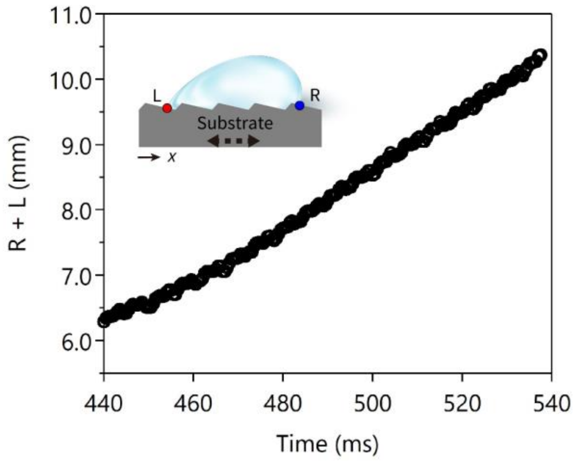

Figure S4. Sum of the droplet position of R and L at frequency of 90 Hz on the sawtooth surface of α = 13 degrees during droplet transport in the synchronized regime.

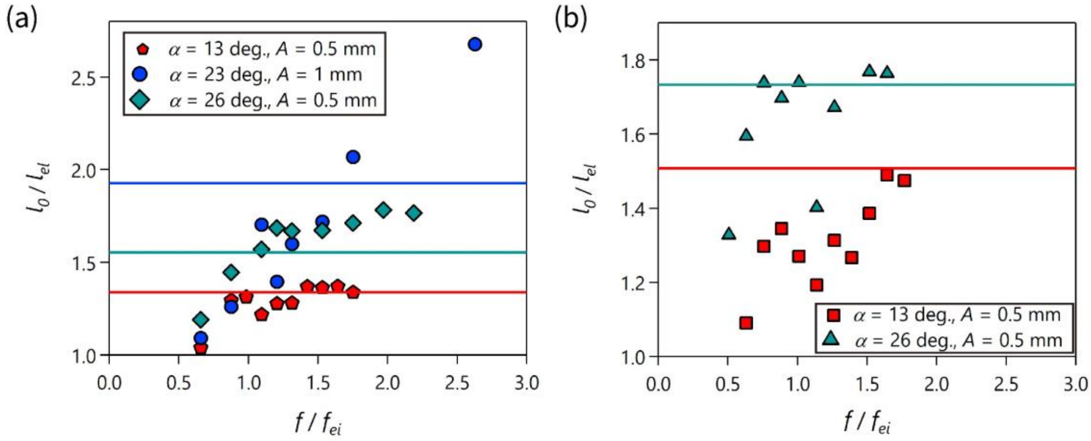

Figure S5. Non-dimensional elongation length (l_0/l_{el}) of droplet to non-dimensional frequency (f/f_{ei}, $f_{ei}=\omega_r/2\pi$). The values of l_0/l_{el} were obtained by averaged length of footprint (R-L, shown with black triangles in Fig. 2. in the main text, for example) through 5 cycles of oscillation l_{el} divided by the corresponding initial value of l_0 before oscillation starts. Solid lines show the expected maximum value of $(l_0 + 2A)/\ l_0$. Each plot of (a) and (b) corresponds to the droplet volume of 6 µl and 2 µl respectively.

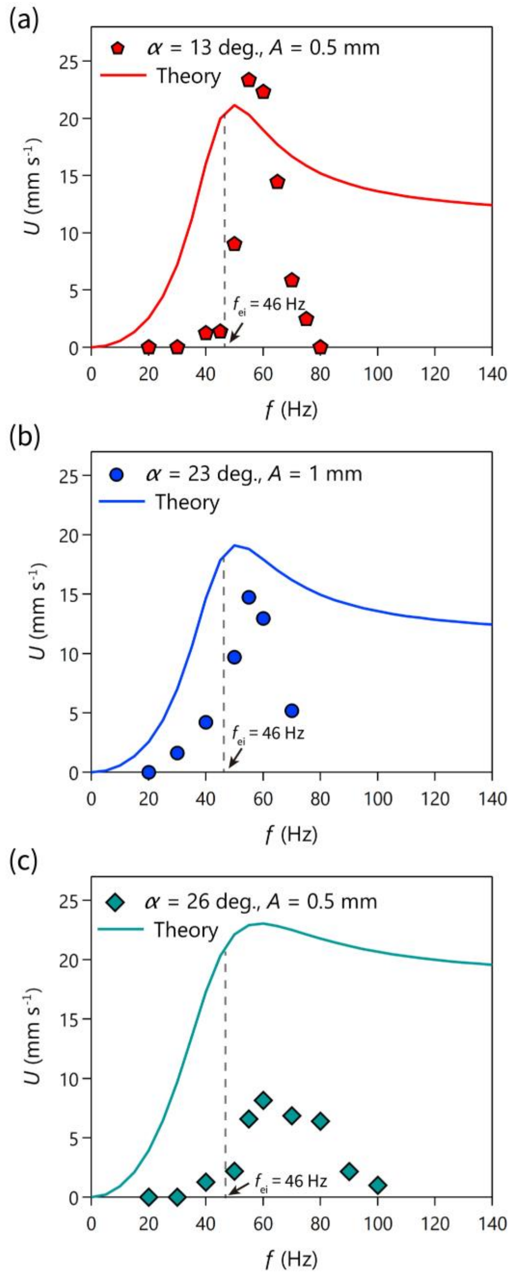

Figure S6. Translational speed of water droplet over the sawtooth surface depending on the driving frequency with 6 μl droplet respectively for (a) α = 13 degrees, (b) α = 23 degrees, and (c) α = 26 degrees structures. Solid lines show theoretically derived translational speed from equation 6 in the manuscript corresponding to each experimental condition.

Table S1. Roughness factor of S_d and S_u for down and up directions respectively calculated from the Eqs. S9 and S10.

Geometry	S_d	S_u
α = 13 deg.	1.22	1.59
α = 23 deg.	1.59	1.72
α = 26 deg.	1.65	3.33

$$S_u = \left(\frac{\sin\sigma}{\sin(\alpha+\beta)}\frac{\sin(\theta_g+\alpha)}{(\cos\theta_e-\cos(\theta_g+\alpha))} + \frac{\sin\sigma}{\sin(\alpha+\sigma)}\frac{\sin(\theta_g-\sigma)}{(\cos\theta_e-\cos(\theta_g-\sigma))}\right)\frac{\cos\theta_e-\cos\theta_g}{\sin\theta_g} \tag{S9}$$

$$S_d = \left(\frac{\sin\alpha}{\sin(\alpha+\beta)}\frac{\sin(\theta_g+\sigma)}{(\cos\theta_e-\cos(\theta_g+\sigma))} + \frac{\sin\sigma}{\sin(\alpha+\sigma)}\frac{\sin(\theta_g-\alpha)}{(\cos\theta_e-\cos(\theta_g-\alpha))}\right)\frac{\cos\theta_e-\cos\theta_g}{\sin\theta_g} \tag{S10}$$

Table S2. Roughness factor of S_d and S_u for down and up directions respectively calculated from the Eqs. S9 and S10.

Geometry	S_d	S_u
α = 12 deg., β =115 deg., σ = 53 deg.	1.20	1.57
α = 14 deg., β =115 deg., σ = 51 deg.	1.24	1.60
α = 25 deg. β =87 deg. σ = 68 deg.	1.61	3.37
α = 27 deg. β =87 deg. σ = 66 deg.	1.68	3.30

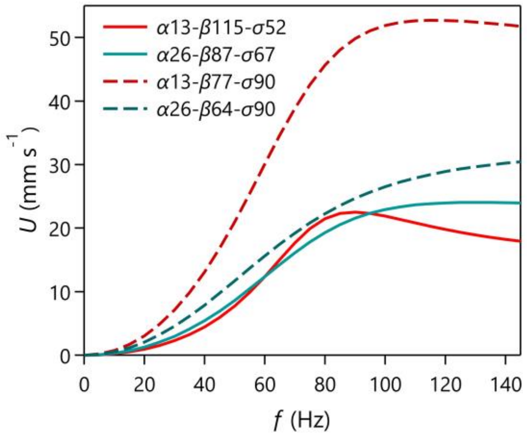

Figure S7. Solid lines show theoretically derived translational speed of 2 μl water droplet from Eq. (6) in the manuscript corresponding to the geometries of α13 and α26 structures used in the experiments. Dashed lines illustrate theoretical speed with geometry of conventional sawtooth shape with sigma of 90 degrees when α is same values respectively.

Movies (mov) of oscillating droplets

Movie S1: A pure water droplet with volume of 2 μl on an asymmetric sawtooth shaped structure with geometry of α = 13 deg., β = 115 deg., σ = 52 deg. and p = 1.7 μm. The surface structure was vibrated at f = 90 Hz with A = 0.5 mm, and the droplet travels to the down direction (relative to the surface geometry) finally reaching at 23 mm/s. The playback speed is × 0.2 compared to the real time. This movie corresponds to Figures 1b.

Movie S2: Slower playback speed of Movie. S1 to catch the detailed droplet motion which corresponds to Figure 2d. The playback speed is × 1/250 compared to the real time.

Movie S3: A pure water droplet with volume of 2 μl on an asymmetric sawtooth shaped structure with geometry of α = 13 deg., β = 115 deg., σ = 52 deg. and p = 1.7 μm. The surface structure was vibrated at f = 130 Hz with A = 0.5 mm, and the droplet travels to the down direction (relative to the surface geometry) finally reaching at 3.4 mm/s. The playback speed is × 0.2 compared to the real time. This movie corresponds to Figure 2e partially at the latter time between 5 s and 6 s of the movie.

Movie S4: A pure water droplet with volume of 2 μl on an asymmetric sawtooth shaped structure with geometry of α = 13 deg., β = 115 deg., σ = 52 deg. and p = 1.7 μm. The surface structure was vibrated at f = 140 Hz with A = 0.5 mm. The droplet does not travel over the surface with elongated footprint length. The movie was taken with CMOS image sensor and the playback speed is × 1 compared to the real time.

Movie S5: A pure water droplet with volume of 6 μl on an asymmetric sawtooth shaped structure with geometry of α = 23 deg., β = 112 deg. σ = 45 deg. and p = 0.82 μm. The surface structure was vibrated at f = 55 Hz with A = 1 mm, and the droplet travels to the down direction (relative to the surface geometry) finally reaching at 15 mm/s. The playback speed is × 1/15 compared to the real time. This movie corresponds to Figure 3c partially at the middle time between 2 s and 3 s of the movie.

Movie S6: A pure water droplet with volume of 6 μl on an asymmetric sawtooth shaped structure with geometry of α = 23 deg., β = 112 deg. σ = 45 deg. and p = 0.82 μm. The surface structure was vibrated at f = 120 Hz with A = 1 mm, and the droplet travels to the up direction (relative to the surface geometry) finally reaching at 5.2 mm/s. The playback speed is × 1/15 compared to the real time. This movie corresponds to Figure 3c partially at the middle time between 2 s and 3 s of the movie.

Movie S7: Various volume of droplets lying on an asymmetric sawtooth shaped structure with geometry of α = 13 deg., β = 115 deg., σ = 52 deg. and p = 1.7 μm. The total surface area is 15 mm × 15 mm. The surface structure was vibrated at f = 90 Hz with A = 0.25 mm for one second including the rising and falling time of the vibration and paused. Then the surface was vibrated in the same way for one point five seconds.

Movie S8: Various volume of droplets lying on an asymmetric sawtooth shaped structure with geometry of α = 13 deg., β = 115 deg., σ = 52 deg. and p = 1.7 μm. The total surface area is 15 mm × 15 mm and the surface structure was vibrated at f = 90 Hz with A = 0.5 mm. Only 2 μl droplet move to down direction rapidly at U > 20 mm s^{-1} while the biggest

droplet with volume of 6 μl is stationary. Some of smaller droplets with volume of 1 μl moves to down very slowly except the one droplet pinned at the top edge of the surface structure.

Movie S9: Various volume of droplets lying on an asymmetric sawtooth shaped structure with geometry of α = 13 deg., β = 115 deg., σ = 52 deg. and p = 1.7 μm. The total surface area is 15 mm × 15 mm and the surface structure was vibrated at f = 40 Hz with A = 0.5 mm. Only the biggest 6 μl droplet move to down direction at low speed of $U \sim$ 1 mm s^{-1} while the other smaller droplets with volume of 1-2 μl are stationary. Once the 6 μl droplet merge with the 2 μl droplet in front of, the merged droplet with volume of 8 μl stops to move.
